# The future of AI in critical care is augmented, not artificial, intelligence

**DOI:** 10.1186/s13054-020-03404-5

**Published:** 2020-12-02

**Authors:** Vincent X. Liu

**Affiliations:** 1 Division of Research, Kaiser Permanente, 2000 Broadway, Oakland, CA 94612 USA; 2 The Permanente Medical Group, Oakland, CA USA

The field of AI—artificial intelligence—has seen tremendous success over the past decade. Today, AI touches billions of lives each day through voice and text processing, computer vision, prediction algorithms, video games, and much more. Naturally, there has also been enormous interest in applying AI to health care and, in particular, to data-rich environments like the intensive care unit. Early examples of AI in healthcare and critical care have already shown great promise [1], but also raise concerns that can be mitigated with preparation and foresight [2–4].

Recently, I put my own life into the hands of AI: it nearly killed me and, later, it also saved me. This harrowing experience was a potent reminder for me, an AI practitioner, that we must work to ensure this technology’s formidable capabilities are used to produce ‘augmented’, rather than just ‘artificial’, intelligence. Augmented intelligence places clinicians and ultimately patients, rather than algorithms, at its center. Where we successfully bridge the interface of clinician and machine intelligence, we have vast potential to make healthcare more effective, efficient, and sustainable. This will also ensure that health AI is safe, reliable, and equitable for all patients.

In December, I found myself driving a Tesla electric car from Seattle to the Bay Area. With its highly touted AI—the car’s technology deploys sensors, computer vision, and deep learning to drive under its own guidance—having logged billions of driving miles, I anticipated a seamless transition between myself and the vehicle. What I experienced instead was a life and death struggle for control. After activating the AI, the car accelerated and took control of the wheel. Surprised, I searched for a way to disengage the technology. My first slight turn of the wheel proved ineffective. A more forceful attempt was interpreted by the vehicle as a course deviation. The AI immediately countered my turn, hurtling us toward a concrete barrier. Back and forth, the car swerved as the AI and I fought for control. Only after coming to a full stop on a busy highway was I finally able to regain control.

In the end, the AI worked precisely as it was designed, following its algorithms. Yet, in succeeding in its task, it failed to produce a safe driving environment for its user. Although rare, similar events have contributed to fatal car and airline accidents. In a recent example, aviation software algorithms left pilots struggling to take control of their aircraft, ultimately contributing to hundreds of deaths. Inexperience and a lack of training magnified the danger induced by AI-driven actions.

The object lesson for critical care is that we must ensure that our clinicians are prepared to effectively use future AI tools. This will require careful design of the human–machine intelligence interface and training in the interpretation of algorithmic outputs. Today, we contextualize laboratory data using clinical intelligence. While a lactate, troponin, or creatinine value of 5 are all poor prognostic signs, it is our clinical judgment, not a single laboratory value, which guides which patients will receive vasopressors or inotropes, cardiac catheterization, or dialysis, respectively. In the future, we will have to contextualize complex streaming AI outputs. We must be prepared to use these tools, aware that they will occasionally produce outlandish, and even frankly dangerous, recommendations.

Trusting AI recommendations presents another key challenge. Explainable AI—the methods that peer inside deep learning’s ‘black box’ [5]—will help to garner clinician trust. Ultimately, however, AI explainability may be overrated: I cannot explain how my lab measures sodium values and, yet, I act on them daily. With experience, I have gained trust in my car’s AI. On a steady road with free-flowing traffic, the technology performs amazingly well. Under congested or uncertain conditions, my trust wanes and I disengage the technology. Supporting a similar learning curve in critical care will maximize AI’s benefits and minimize its attendant risks.

Perhaps the most vexing challenge in using AI tools will be addressing the faults embedded within them. Algorithms are designed to relentlessly achieve a specific objective; this explains why gaming AI agents may break rules or ‘cheat’ their way to the most efficient solutions [6]. In healthcare, some algorithms succeed by finding hidden clues, rather than true clinical signals, to optimize performance. This can make AI tools brittle: minor changes in data, like a single pixel in an image, can cause them to fail [7].

Finally, algorithms are trained using existing data and, thus, encode prior decisions and biases within them. This is not a new problem—we are grappling with biases revealed within well-established clinical tools [8, 9]—but one which will be magnified as AI tools reach the bedside. To address this, we must ensure that more representative datasets are available for AI development and that we pre-identify systemic biases to avoid unintended consequences. We will also need rigorous prospective studies to assess which AI tools truly improve patient outcomes [10, 11].

Although my car’s AI had brought me to the brink of demise, it also later saved me. As my drive home grew lengthy, stopping every 3 h to recharge the car’s batteries, I began nodding off at the wheel. When called upon again, the car’s AI worked precisely as designed, augmenting my diminished capabilities and guiding me home safely.

AI has begun to touch every aspect of our lives and it will revolutionize our approach to health and critical care. Undoubtedly, the road ahead has potential hazards. By ensuring that emerging AI tools are designed to produce augmented, rather than just artificial, intelligence, we will secure AI’s greatest benefits for our clinicians and our patients.

## Data Availability

Not applicable.
